# Crystallographic Characterisation of Ultra-Thin, or Amorphous Transparent Conducting Oxides—The Case for Raman Spectroscopy

**DOI:** 10.3390/ma13020267

**Published:** 2020-01-07

**Authors:** David Caffrey, Ainur Zhussupbekova, Rajani K. Vijayaraghavan, Ardak Ainabayev, Aitkazy Kaisha, Gulnar Sugurbekova, Igor V. Shvets, Karsten Fleischer

**Affiliations:** 1 School of Physics, Trinity College, The University of Dublin, Dublin 2, Ireland; dcaffrey@tcd.ie (D.C.);; 2 School of Electronic Engineering, Dublin City University, Glasnevin, Dublin 9, Ireland; 3 Nazarbayev University, Laboratory of Materials Processing and Applied Physics, Nur-Sultan 010000, Kazakhstan; 4 School of Physical Sciences, Dublin City University, Glasnevin, Dublin 9, Ireland

**Keywords:** transparent conducting oxide, TCO, Raman spectroscopy, amorphous oxide, oxide electronics, background subtraction

## Abstract

The electronic and optical properties of transparent conducting oxides (TCOs) are closely linked to their crystallographic structure on a macroscopic (grain sizes) and microscopic (bond structure) level. With the increasing drive towards using reduced film thicknesses in devices and growing interest in amorphous TCOs such as n-type InGaZnO${}_{4}$ (IGZO), ZnSnO${}_{3}$ (ZTO), p-type Cu${}_{x}$CrO${}_{2}$, or ZnRh${}_{2}$O${}_{4}$, the task of gaining in-depth knowledge on their crystal structure by conventional X-ray diffraction-based measurements are becoming increasingly difficult. We demonstrate the use of a focal shift based background subtraction technique for Raman spectroscopy specifically developed for the case of transparent thin films on amorphous substrates. Using this technique we demonstrate, for a variety of TCOs CuO, a-ZTO, ZnO:Al), how changes in local vibrational modes reflect changes in the composition of the TCO and consequently their electronic properties.

## 1. Introduction

Transparent conducting oxides (TCOs) are materials that combine high optical transparency with good electrical conductivity. They see widespread use as transparent contacts in solar cells, touch screens, transparent heaters for anti-fogging windows and contacts in electrochromic windows [1,2,3,4]. In recent years they have also gained attention for their role in the improvement of pixel driving thin-film transistors in displays [5,6].

For most applications requiring high currents, crystalline TCOs such as indium–tin oxide (ITO, In${}_{2}$O${}_{3}$:Sn), fluorinated tin oxide (FTO), and aluminated Zinc Oxide (AZO) are employed. There is, however, growing interest in amorphous TCOs such as n-type a-InGaZnO${}_{4}$ [7,8,9] or ZnSnO${}_{3}$ (ZTO) [10]. The latter offer advantages in higher lateral homogeneity, lower deposition temperature, and a certain stability under mechanical stress during bending [11,12,13,14]. A second development is the use of TCOs as functionalisation or protective layers in hybrid structures [15,16] or functionalised gas sensors [17], where with the advent of atomic layer deposition (ALD) it is possible to grow and employ very thin conformal layers of oxides [18,19,20]. In both of these cases, it is becoming increasingly difficult to assess the relationships between crystallographic order and electronic properties. This is caused by either the lack of any long-range order (amorphous materials) or size broadening due to the reduced scattering volume and small coherent grain size in ultra-thin oxides. All these effects greatly limit the use of X-ray diffraction (XRD), conventionally used for probing crystalline quality. Alternative methods, with larger scattering cross-section and sensitivity to local bond structures, rather than long-range order, are hence sought for. Particularly for recently developed amorphous or non-crystalline p-type TCOs gaining additional information on the local bond structure will be helpful to gain insights on the doping mechanisms [21,22,23,24,25,26,27].

Raman spectroscopy, measuring vibrational (phonon) modes of samples, can provide exactly this insight. As the phonon modes in any material depend on the distance, mass and geometric arrangement of atoms, measuring the energy of the phonon modes reveal information on the crystallographic order, as well as size-related effects within nanostructured material [28,29,30]. The position of the Raman mode reveals indirect information on stoichiometry, while the width of the modes is linked to disorder. In contrast to XRD the local bond order plays an important role and long-range order is not required to obtain a signal. Hence, amorphous structures can also be measured and it is frequently used to characterise amorphous silicon or carbon [31,32,33].

Unfortunately, transparent materials typically exhibit a low Raman cross-section. Their low absorption coefficient for visible light, where most lasers used for Raman spectroscopy operate, leads to overall small signal levels. Secondly, the cross-section is directly proportional to the scattering volume. Hence in very thin films signal levels drop further and all measurements contain a superposition of thin film and substrate Raman signals. If appropriate background subtraction methods are employed (or if the thin film signal is very strong) it is nevertheless possible to analyse even the different vibrational signature of single atomic layers [34,35], or the modes of surface atoms in a different bond arrangement than their bulk counterparts [36].

**Figure 1:**
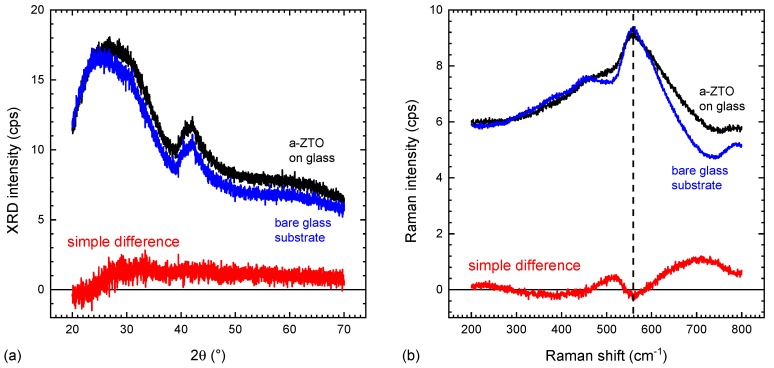
Example of the failings of diffraction-based techniques and simple background subtraction method for Raman spectra of a thin amorphous transparent conducting oxides (TCOs) on glass. (**a**) X-ray diffraction (Bruker D8 Advance, unmonochromised Cu Kα in parallel beam geometry) measured for a bare glass substrate and a 60 nm thick a-ZnSnO${}_{3}$ (ZTO) grown on glass by spray pyrolysis. No discernible signal from the thin film is found; (**b**) Raman spectra for the same samples. It is obvious that there are subtle differences indicating the presence of the thin film. A simple subtraction of a reference measurement on a bare substrate, however, results in a spectrum dominated by residual substrate structures (see dashed line). The film peaks are broader than the glass signal and polynomial background subtraction methods, relying on sharper features of the film compared to the background, will hence fail as well.

In the case that Raman modes are energetically well separated from the bulk modes, the thin film or surface structure can be directly analysed. Likewise if Raman modes of the thin film are sharp, while the bulk substrate shows broad, amorphous structures, automated background subtraction techniques based on polynomial fits or splines can be employed [37,38,39]. However, in the specific case of a transparent, amorphous substrate (i.e., glass, polymers) and a likewise amorphous film (the TCO) this is not the case. The amorphous substrate has very broad features overlapping substantially with the likewise broad signals of the amorphous film. In addition, the broad nature of the amorphous peaks themselves means that inaccurate or inconsistent background subtraction is more likely to result in the creation of “false” structures that are hard to distinguish from those of the actual films. Such a case is illustrated in Figure 1 for spray pyrolysis grown amorphous zinc tin oxide on glass. Thus the reliability of detailed Raman studies on such materials requires the development of a reproducible methodology for background subtraction.

In this paper, we will discuss one specific method which utilises two measurements, taken in a microscope set up at different sample heights (focal point shift). The method is a specific implementation of more generalised depth profiling methods, typically used for 3D-Raman mapping of biological tissues [40,41,42]. We will outline the benefits and drawbacks of the technique, and secondly employ it to demonstrate the method for two materials; a-ZnSnO${}_{3}$ films grown by magnetron sputtering and spray pyrolysis on amorphous glass substrates and ALD grown ZnO:Al on crystalline a-plane Al${}_{2}$O${}_{3}$. In both cases extracted thin-film Raman spectra reveal direct information on the local bond order in the TCOs and can be linked to the film’s electrical and optical properties.

## 2. Focal Shift Background Subtraction

To motivate the use of a vertical focal shift to subtract the substrate Raman signal, we have to briefly discuss the typical scattering volume in a micro Raman setup. While details vary between instruments, it is common practice to focus a laser source through the microscope objective onto the sample surface. By using a mirror or beam splitter, most systems combine the laser optics with a white light source. In this case the sample surface is first inspected through the microscope in normal imaging mode with the image seen either through the ocular or via a camera on screen. Once the white light is adjusted so that the sample surface is in focus, illumination is changed to the laser. Light from the sample is then directed to a monochromator and the Raman spectra are taken from the same area of the sample, where the image was seen previously. The focal point of the laser, and hence imaging area can be as low as typically 1–2 μm in diameter (depending on the objective). We will denote this focal point diameter as $d_{\mathrm{focus}}$, though in reality intensities are distributed in a Gaussian distribution with larger broadening in the *z* direction compared to *x* and *y* as the spot size is typically optimised for high lateral resolution ($d_{x,y}<d_{\mathrm{focus}}$). Numerical values will vary depending on the numerical aperture of the instrument [42]. For our consideration only the *z* dimensions are of importance as only laterally homogeneous thin films are discussed.

**Figure 2:**
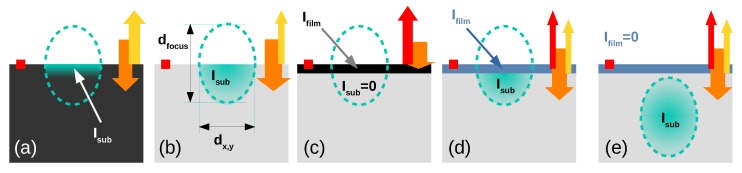
Schematics of the sample volume, where the Raman signal is originated, as well as reflectance losses in a Raman microscope. The dashed ellipsoid indicates the asymmetric focal point of lateral width $d_{x,y}$ and depth distribution $d_{\mathrm{focus}}$. The green, shaded areas indicate the volume generating the substrate Raman signal $I_{\mathrm{sub}}$. (**a**) depicts a bulk absorbing substrate; (**b**) a transparent substrate; (**c**) an absorbing thin film, where no substrate signal occurs; (**d**) a thin film on transparent substrate, creating signal from the film $I_{\mathrm{film}}$ and substrate $I_{\mathrm{sub}}$; and (**e**) the same but with focus shifted into the sample and no film signal. The focal point in Raman spectroscopy is typically determined by focusing on small surface structures as indicated by the red box. The orange arrows depict the incident laser light transmitted into the substrate, yellow arrows the fraction reflected at the substrate surface and red arrows additional reflection by the thin film.

For samples with a large absorption coefficient, a substantial part of the incoming laser light is elastically reflected at the surface. The transmitted part, entering the sample, is then inelastically scattered within the sample creating the Raman signal. The penetration depth of the laser light defines the area actually probed. In strongly absorbing samples the penetration depth can be as low as a couple of nm, while for non-absorbing samples the Raman signal is generated within the entire volume of the focal point. Figure 2 illustrates the situation for a couple of scenarios.

In Figure 2a a strongly absorbing sample leads to signal generated only within the surface area of the sample and the maximum signal is seen when the widest part of the focal point is in the sample surface plane. This is the typical operation mode for a micro Raman setup, where the sample surface is put in focus by obtaining a sharp optical image of small surface imperfections (indicated by the red box in the figure). The largest Raman signal is then observed at this “objective to sample” distance, as the focused laser has the widest intersection with the sample surface. The Raman scattering volume is then given by the diameter of the laser focal point and the penetration depth of the specific sample. For absorbing thin films, where the penetration depth is smaller than the film thickness, the measured Raman spectra then directly relates to the thin film Figure 2c.

However, for transparent samples, with a low absorption coefficient and hence a penetration depth significantly larger than the diameter of the focal point, the situation changes. The Raman signal is now generated in the *entire volume* of the focal point. As a consequence the strongest substrate signal is no longer seen when the sample surface is in focus as depicted in Figure 2b. Instead, it will be visible once the focal point is shifted into the bulk of the sample (Figure 2e). If we now want to measure a transparent thin film as shown in Figure 2d, the measured Raman signal is a linear combination of the thin film ($I_{\mathrm{film}}$) and substrate signal ($I_{\mathrm{sub}}$). The ratio between both contributions depends primarily on geometric factors such as the thin film thickness $d_{\mathrm{film}}$ with respect to the focal point diameter $d_{\mathrm{focus}}$. Once the film is very thin ($d_{\mathrm{film}}\ll d_{\mathrm{focus}}$) the scattering volume within the substrate will not substantially deviate from the substrate-only case. It would be tempting to now simply measure a blank substrate and subtract this measurement from the measurement of the sample with the thin film. Unfortunately for the case of a transparent or semi-transparent film on a transparent substrate, e.g., TCO or transparent device on glass or plastic, this results in a significant error, as the difference in refractive index between the material and substrate adds an additional reflective loss at the material/substrate interface which is not observed in the case of a bare substrate (see Figure 1). As a consequence a larger fraction of the incident laser light is reflected and the substrate contribution to the overall measured signal is reduced. A second drawback is that measuring the substrate separately can create additional systematic errors once the substrates themselves are not homogeneous. While lateral inhomogeneities are typically no issue for modern glass substrates, variations between substrate batches can be when measuring very thin films. If the magnitude of the thin film signal is comparable to variations within the substrate or between batches of substrates, a subtraction of a single substrate measurement can create problems. Specifically on polymer substrates with larger inhomogeneities this issue is important.

For these cases, it is possible to use a different approach. By measuring with the thin film surface in focus ($I_{\mathrm{ff}}$) and subsequently by moving the sample upwards by a distance larger than the focal point diameter (see Figure 2d,e) one can create *two datasets*, where the thin film signal can be extracted using a single geometric factor only. The measurement with the focal point within the substrate ($I_{\mathrm{sf}}$) gives the substrate only signal measured in the same (lateral) area as the first measurement including the thin film and crucially using the same overall sample stack reflectivities for the incident laser light. Therefore, subtracting the latter from the first measurement ($I_{\mathrm{ff}}-cI_{\mathrm{sf}}=I_{\mathrm{film}}$) gives a more reliable thin film Raman signal ($I_{\mathrm{film}}$). The factor *c* is now a pure geometric factor linking the different scattering volumes for the substrate in both measurements. If the thin film thickness is negligible the factor would simply be (c=1/2) as twice the substrate volume is probed in the case depicted in Figure 2d. If the film thickness can not be neglected anymore, but remains smaller than the focal point diameter we can use the following first-order approximation:

(1) $$c=\frac{d_{\mathrm{focus}}-d_{\mathrm{film}}}{2d_{\mathrm{focus}}}.$$

**Figure 3:**
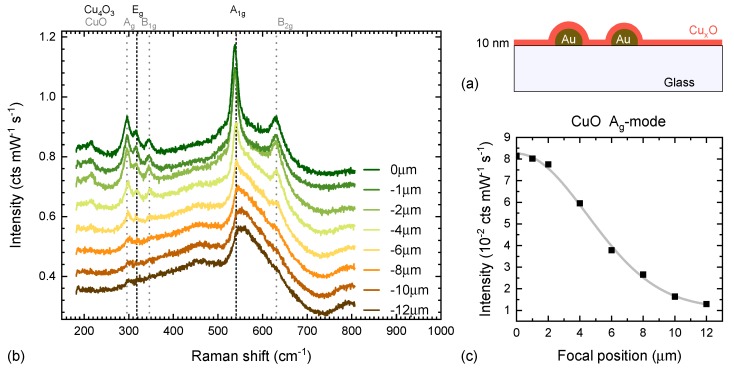
(**a**) Sample geometry of the Cu${}_{x}$O/glass sample with Au nanoparticle (NP) dimers; (**b**) Raman spectra of the sample moving the focal point into the sample. The 0 height is the position where the thin film signal was found to be strongest. By shifting the sample by 12 μm upwards, no CuO Raman modes remain and only the glass Raman spectra is observed; (**c**) Intensity of the CuO A${}_{g}$ Raman mode at 300 cm${}^{-1}$ as a function of focal depth, as well as a fit of the line shape using a Gaussian distribution.

In reality, *c* will be slightly smaller due to the radial symmetry and Gaussian shape of the focal point. For film thickness comparable to $d_{\mathrm{focus}}$ we overestimate the substrate contribution by using (1). In the thin-film limit this error vanishes as *c* approaches exactly 1/2, even for Gaussian beam profiles. The simple method can be further improved by deconvoluting spectra taken at multiple depths and measured instrumental beam profiles (see Figure 3c).

## 3. Proof of Concept and Measuring the Instrumental Constant $d_{\mathrm{focus}}$

To employ the discussed method of background subtraction, a reproducible z−positioning of the samples is required. All measurements shown here have been performed on a JY Horiba LabRAM800 Confocal Micro-Raman Spectroscopy System with motorised positioning stages. The excitation wavelength was 488 nm, power ∼10 mW, and a typical integration time of 5 min.

In order to employ (1), the effective focal depth, as well as the film thickness has to be known. $d_{\mathrm{focus}}$ is an instrumental constant, which will depend on the microscope objective, as well as the incident laser wavelength. In order to estimate the latter for the instrument used here, a reference test sample has been grown showing a comparably strong thin-film oxide Raman spectra. (see Figure 3)

At the same time, the reference sample should have a comparable geometry and substrate to the transparent, amorphous samples of interest. To fulfill both conditions we have grown a very thin film of Cu${}_{x}$O on glass by spray pyrolysis. Copper acetyl acetonate precursor has been diluted in methanol (0.05 M solution) and sprayed using an ultrasonic nebuliser on a glass slide kept at 300${}^{\circ }C$. The sample thickness was confirmed by XRR to be 10 nm with 3 nm roughness. In order to enhance the sample’s Raman signal a small area of the glass sample was covered in plasmonic gold dimers with the average height of 40 nm, 100 nm diameter and gap between adjacent nanoparticles (NPs) of 100 nm. The NP dimer synthesis procedure is discussed elsewhere [43]. The NPs or Cu${}_{x}$O thickness have not been optimised for maximum Raman enhancement, but are sufficient to aid the process in creating a strong Raman signal from the very thin film. Figure 3 shows a schematic cross-section of the sample, as well as Raman spectra taken at different z−positions of the sample.

The Raman spectra of this sample shows a mixture of CuO and Cu${}_{4}$O${}_{3}$ phases. The Cu${}_{4}$O${}_{3}$-A${}_{1g}$ mode at 538 cm${}^{-1}$ was found to be strong enough to be seen even in 1 s integration time spectra, allowing for conventional maximisation of the thin film Raman signal by manually focusing while continuously measuring. The point of the strongest thin film signal would normally then be set to be the reference height of 0 μm. As seen in Figure 3, the strongest mode, unfortunately, coincides with a structure of the glass background. The analysis of how the thin film Raman intensity changes with focal depth (see Figure 3c) was hence done on a different peak, namely the CuO A${}_{g}$ mode at 300 /cm^−1^. As expected, the thin film signal disappears and only the substrate Raman signal is measured once the focus is moved into the substrate. Fitting the change in amplitude as a function of focal depth, using a Gaussian distribution resulted in an estimation of the focal depth of 8 μm (using the full-width half maximum of the distribution as the measure). This test also shows that for a focal depth of 12 μm almost no thin-film signal remains, allowing us to treat it as a “substrate only” spectrum.

**Figure 4:**
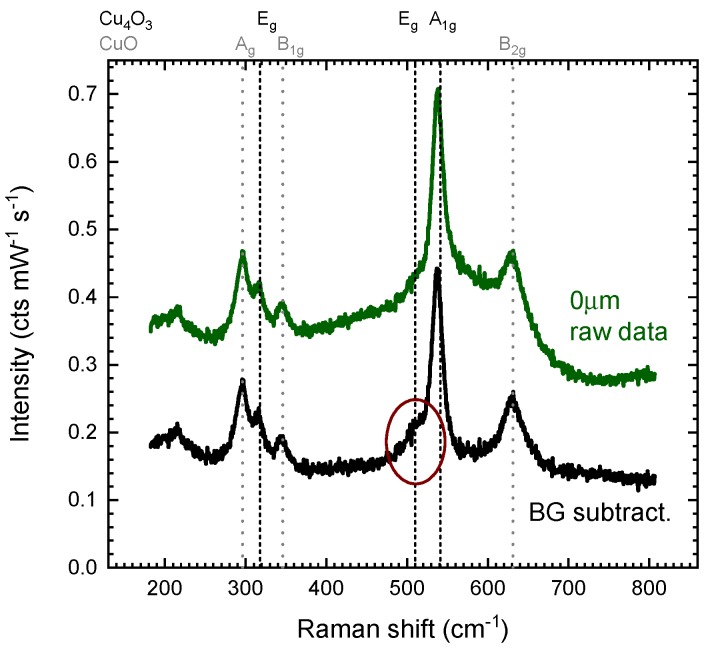
Raw data and background-subtracted data of the 10 nm Cu${}_{x}$O sample, using the described focal shift method. In subtracted data Raman modes of the copper oxide are seen as more symmetric and smaller modes (red circle) can be identified. The effect of the background subtraction is limited, as the thin film signal is very strong for this reference sample.

Figure 4 compares the raw measurement at 0 μm with the background-subtracted spectra using the measurement at −12 μm as substrate signal and c=2, as the effective focal point size of ≈ 8 μm is much larger than the film thickness of 10 nm. It can be seen that for this specific sample the background subtraction is not crucial as all main features are already seen in the raw data. However in the subtracted spectra, the modes in the region of a strong glass signal are now seen more symmetric (e.g., CuO B${}_{2g}$ mode at 631 cm^−1^), and weak modes such as the Cu${}_{4}$O${}_{3}$ E${}_{g}$ mode at 510 cm^−1^ can be assigned with greater confidence (red circle in Figure 4).

## 4. Example Amorphous Zinc Tin Oxide

With the feasibility of the approach confirmed, we can apply the methodology to a set of amorphous zinc tin oxide (a-ZTO) samples grown with different techniques and conditions. In doing so we can test the capability of the background subtraction technique to consistently separate the Raman signal from the amorphous glass background. We will also demonstrate a use for this information by using the differences in local bond order to determine the a-ZTO composition by comparison to previously grown films. This indirect analysis of the composition was later confirmed using X-ray photo-electron spectroscopy a direct compositional analysis technique. We have previously employed a more simplistic background subtraction method (constant c=1/2) to evaluate the different bond order in magnetron sputtered a-ZTO [44]. It was found that there are characteristic broad modes at ≈500 and 650 cm${}^{-1}$, with changes in their relative amplitude as function of Zn content *x*. Here we use *x* to denote the relative Zn content according to the relationship ${\left(\mathrm{ZnO}\right)}_{x}{\left({\mathrm{SnO}}_{2}\right)}_{1-x}$ (0<x<1). In this notation stoichiometric ZnSnO${}_{3}$ is found for x=0.5 (Zn:Sn = 1:1).

**Figure 5:**
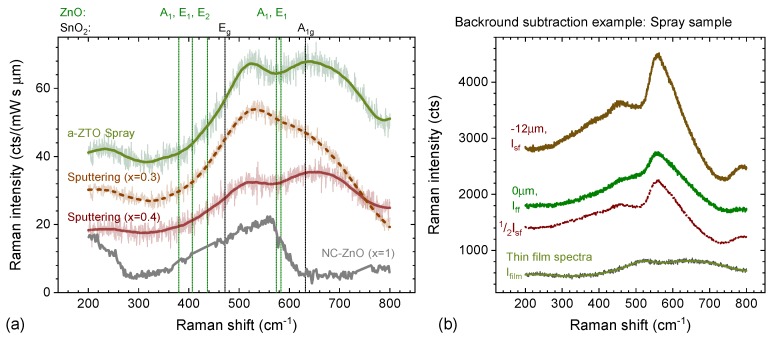
(**a**) Background subtracted Raman spectra of a-ZTO films grown by magnetron sputtering and spray pyrolysis. The Raman spectra are dominated by two broad modes at 500 and 650 cm${}^{-1}$. Higher tin content leads to an increase of the amplitude of the 500 cm${}^{-1}$ mode. The spectra of the spray pyrolysis sample is very similar to the one of the sputtered sample with x=0.4 suggesting it has a similar Zn content. The gray spectra is an Al-doped ZnO sample with high Al content (8%) indicative of nanocrystalline ZnO (for more details see Section 5); (**b**) example of raw data ($I_{\mathrm{ff}},I_{\mathrm{sf}}$) and background-subtracted data $I_{\mathrm{film}}$ for the Spray pyrolysis sample. In contrast to the CuO${}_{x}$ case raw spectra are dominated by the glass substrate Raman spectra and only subtle changes in line shape indicate the presence of the film.

As the first step, we utilised this methodology to examine those films. Figure 5 shows the Raman spectra of two magnetron grown samples with x=0.3 and 0.4. Both samples were grown by reactive sputtering in Ar and O${}_{2}$ from metallic Sn targets and oxide ZnO targets onto glass substrates at 300 ${}^{\circ }C$. Further details on growth conditions are found in [44]. Despite their distinctly different composition both samples are conductive with conductivities in excess of σ≈150 S/cm^−1^.

In the second step, we have evaluated samples grown by spray pyrolysis. Using methanol as solvent, solutions with 0.05 M Zinc chloride and 0.02 M tin(II)2-ethylehexanoate were sprayed at 450 ${}^{\circ }C$ with 2 mL/min fluid spray rate and 15 L/min nitrogen carrier gas using an air blast nozzle. The nominal precursor ratio for the initial test was estimated using known growth rates of plain ZnO and SnO${}_{2}$, aiming for a Zn content within the final ZTO film in the range of 0.3–0.5. While substantially lower growth temperatures are desirable from a device perspective, using these specific precursors, the higher temperature was required to achieve as grown conductive films.

All a-ZTO films are nominally undoped with carriers only generated by intrinsic defects. The as grown conductivity of films deposited in these conditions is ≈ 110 S/cm^−1^, comparable to that of magnetron grown films. Raman spectra of the spray pyrolysis film (see Figure 5) show the same broad doublet. Despite the higher growth temperature, there are no indications of Raman modes of either ZnO, nor SnO${}_{2}$ being present (see reference positions from [45,46] indicated in Figure 5). Alternative intermediate tin oxide phases [47] are also not observed. Assuming the previous findings on the shape of the Raman spectra can be directly applied to samples grown at higher temperatures by spray pyrolysis, the spectra indicate that the Zn content in this specific spray sample is close to x=0.4. Indeed X-ray photo-electron (XPS) measurement reveal a value of x=0.42. This suggests three things:

- As suspected, the 5:2 molarity of Zn and Sn precursors is not directly transferred into the stoichiometry of the grown oxide, found to be tin rich.
- The spray pyrolysis recipe requires further optimisation, as in magnetron samples the best conductivity in oxygen-rich growth conditions (using SnO${}_{2}$ targets) was found to be closer to x=0.27. [44]
- Most importantly the background subtraction method, applied to only 50–100 nm thin amorphous films on glass, allows for the extraction of thin-film spectra good enough to qualitatively estimate the stoichiometry of the film. This will be invaluable in further optimisation of the spray process, as measurement times are significantly shorter for Raman spectroscopy compared to XPS.

As shown above Raman spectra on a-ZTO show broad, yet distinct Raman modes at 500 and 650 cm${}^{-1}$. It is unclear at this stage what is the microscopic origin of these modes, other than that an increase in amplitude of the 500 cm${}^{-1}$ mode is observed for a higher tin content. In the same spectral range a structure can be observed in nanocrystalline samples of tin-free nanocrystalline ZnO. However, given the fact that in a-ZTO films this structure increases when reducing levels of ZnO it is likely coincidental. This will be discussed further in Section 5. It is also not possible to say if the increased intensity of this structure is due to a direct connection of the 500 cm${}^{-1}$ mode to an increased number of Sn−O bonds, or a change in the Raman cross-section due to changes in i.e., defect induced absorption. While none of the modes coincide with those of crystalline SnO${}_{2}$ or ZnO, we can not exclude the possibility of phase-separated amorphous or nanocrystalline areas are responsible for the Raman modes at this stage. In general Raman spectra of amorphous materials are extreme cases of size induced broadening, which occurs once the effective crystal size is small enough that Raman spectra are not limited to phonon modes at the Γ point, but average a larger fraction of the Brillouin zone [48]. Depending on the dispersion of the phonon modes, shifts can either be to higher phonon energies (minimum at Γ) or lower phonon energies (maximum at Γ). For ZnO one expects a blue shift for all modes around 400 cm${}^{-1}$, but a redshift for the $A_{1},$ and $E_{1}$ around 580 cm${}^{-1}$ [49,50]. For SnO${}_{2}$ no significant shifts in the $A_{1g}$ mode are expected as there is little dispersion in the mode [51]. The broad structure at 650 cm${}^{-1}$ is inconsistent with either and therefore a unique mode of a-ZTO involving both Sn and Zn atoms. Indeed similarities are seen with Raman spectra of a Zn${}_{2}$SnO${}_{4}$ spinel phase seen in high-temperature annealing [52].

While Raman spectroscopy provides insights into the average bond structure, these early results also indicate that the bond structure only indirectly contributes to the conductivity, as samples with distinctly different compositions and hence bond structure can show similar conductivities. Table 1 summarises the electrical and optical properties for the specific samples shown in Figure 5. A more detailed analysis of the behavior of magnetron sputtered ZTO is found in [44]. While not confirming conduction directly, the Raman data can identify local bond structures capable of maintaining sufficient mobility and hosting the required carrier generating intrinsic defects.

## 5. Example Ultrathin Nanocrystalline Zinc Oxide

In order to exclude the possibility of amorphous ZnO regions contributing to the Raman spectrum of ZTO we can investigate the Raman spectra of nanocrystalline ZnO. We employ the same background subtraction method on a set of aluminum-doped samples (ZnO:Al) grown by atomic layer deposition (ALD) on a-plane sapphire wafers. The sample thickness varied from 50 to 70 nm depending on Al content and sample texture. Details of the growth conditions and film properties are described elsewhere [53]. The use of ALD allows for very low growth temperatures (here 250 ${}^{\circ }C$), while maintaining high conductivity of the TCO layers. Of importance here is that X-ray diffraction (XRD) measurements of these films reveal an amorphisation of the ZnO crystal structure above 6% Al content [53]. Diffraction patterns and all film thicknesses have been measured with a Bruker D8 Discover, using a monochromised Cu-Kα source (Goeble mirror and Ge double bounce monochromator). Samples were aligned to the a−plane Al${}_{2}$O${}_{3}$ substrate peak.

**Figure 6:**
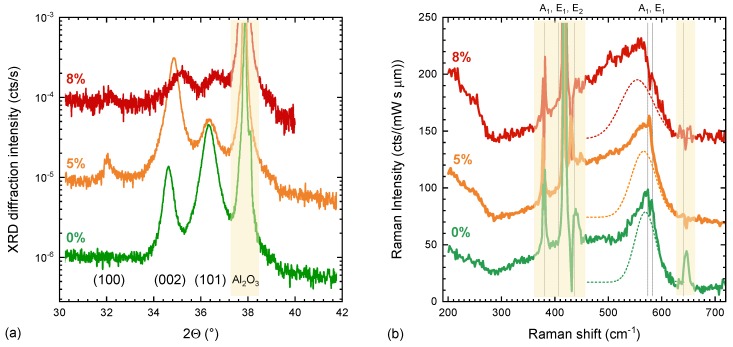
(**a**) X-ray diffraction of atomic layer deposition (ALD) grown ZnO and ZnO:Al samples with increasing Al content; (**b**) background subtracted Raman spectra of the same samples. Regions with strong substrate contributions have been shaded. The broad ZnO response was deconvoluted for each sample by two broad Gaussian peaks. For clarity only the deconvoluted $E_{1}$ mode is shown (dashed line)

Figure 6 shows the Raman spectra and corresponding XRD measurements for undoped, 5% and 8% doped ALD grown ZnO:Al.

The sample with 8% doping shows a strong reduction in the XRD signal, accompanied by a strong shift of peak positions towards higher scattering angles (smaller d-spacing). The (002) reflex for instance shifts from 34.6${}^{\circ }$ to 35.2${}^{\circ }$, but more importantly it substantially broadens and reduces in intensity. Applying the Scherrer equation to estimate the coherent domain size for this sample results in a value for the domain size of ≈8 nm. It is worth highlighting here that the films are not amorphous but instead are nanocrystalline, having crystallographic domains on the scale of a few nanometers. Indeed it is difficult to synthesise amorphous ZnO as has been recently discussed from a theoretical point of view [54]. Experimental methods have previously used large amounts of either Sn alone [55], or Sn, and Ge [56] to reach a fully amorphous zinc-rich ZnSnO${}_{3}$ based material. While not fully amorphous, by comparing the spectra of the nanocrystalline ALD ZnO:Al films to those of higher crystallinity we can gain a better idea of the effects of amorphisation on the Raman spectra. Undoped ZnO samples, with domain sizes of up to 25 nm, show a broad mode at 570 cm${}^{-1}$ (Γ = 38 cm${}^{-1}$ corresponding to longitudinal $E_{1}$ and $A_{1}$ modes found at 583 and 574 cm${}^{-1}$ in bulk ZnO. The red shift and broadening is already consistent with the nanocrystalline nature of the films [57]. It is also consistent with expected size-related shifts from the perspective of the phonon dispersion [50]. The typically stronger $E_{2}$ mode at 438 cm${}^{-1}$ coincides with strong substrate modes in the a-plane sapphire, and, even utilising the background subtraction method, they can not be reliably measured (shaded areas in Figure 6). The relative strength of the $E_{1}$ and $E_{2}$ modes strongly depends on the ZnO orientation, incident laser wavelength, crystalline size, but also thin film texture, which also changes in our case. For example, ZnO nanorods grown on sapphire show substantially stronger $E_{1}$ modes as well [58]. For the samples with even smaller domain size (5% and 8% doping), the $E_{1}$ mode further broadens and redshifts to 566 cm${}^{-1}$ (Γ = 46 cm${}^{-1}$) and 554 cm${}^{-1}$ (Γ = 62 cm${}^{-1}$) respectively. In all cases the anisotropic line shape was accounted for by fitting a second much broader mode around 420–470 cm${}^{-1}$ with Γ>100 cm${}^{-1}$. The position of the mode is consistent with a strong size-related shift of any of the $A_{1},E_{1},$ or $E_{2}$ modes around 400 cm${}^{-1}$ as these have a strong dispersion [50]. The overlap with the residual substrate modes in this area makes a quantitative comparison of this broader mode impossible. However, there is a stark similarity of the Raman spectra found here with the calculated phonon density of state of amorphous ZnO as reported in [57]. Particularly the spectra of the 8% sample can already be interpreted as amorphous ZnO, with two very broad structures at 554 cm${}^{-1}$ and ≈480 cm${}^{-1}$, which can be interpreted to be related to two different Zn−O bond length predicted to be present in a-ZnO [54].

The main finding at this stage is, that while there is qualitatively a similar two-mode structure seen in the a-ZTO (500 cm${}^{-1}$ and 650 cm${}^{-1}$), a-ZnO modes are found at fundamentally different positions (480 cm${}^{-1}$ and 554 cm${}^{-1}$). This proves that the modes seen in the ZTO samples with varying Sn content are indeed reflective of a distinct bond arrangement in ZTO, rather than a superposition on a-ZnO and a-SnO${}_{2}$ modes.

## 6. Conclusions

We have shown that Raman spectroscopy can be a useful tool to assess the local bond order in amorphous TCOs or crystallinity in very thin crystalline TCOs grown on amorphous samples such as glass. The key point is the use of an appropriate background subtraction to reliably distinguish between the substrate and thin-film signal. Our method can be used for films on plain glass samples and does not require specially prepared substrates to enhance the thin film signal by i.e., plasmonic or surface-enhanced resonance. As such the method is therefore specifically useful in material screening experiments, where substrate costs have to be low and substrate temperature stability needs to be high to allow screening of wide processing windows.

We have demonstrated the feasibility of the method for selected test cases such as Cu${}_{x}$O on glass and a-ZnSnO${}_{3}$ on glass and sapphire, as well as employed the method to analyse Raman spectra of a-ZnSnO${}_{3}$ on glass and thin ZnO:Al on Al${}_{2}$O${}_{3}$. Using the technique we were able to demonstrate that spray pyrolysis grown a-ZTO has, as expected, a similar local bond configuration than magnetron sputtered films, allowing for a much faster growth optimisation in terms of testing the Zn/Sn ratio in this complex ternary material. By comparison to nanocrystalline ZnO grown by ALD we could also conclude that the distinct Raman spectra of a-ZnO are not a simple superposition of ZnO and SnO${}_{2}$ like bond arrangements but show a doublet of broad modes below and above the energy of an amorphous ZnO mode.

## Figures and Tables

**Table 1:** Summary of electrical and optical properties of selected ZTO samples shown in Figure 5. The growth method, ZnO content *x*, specific sample thickness *d*, conductivity σ, as well as average transmission *T*, reflection *R*, and T+R in the range from 1–3 eV are listed. All values include the substrate. The relatively low transmission is caused by high reflectivity by interference for the specific film thickness [24]. The film thickness was measured by X-ray reflection. Within the error (±1%) no significant absorption was found in this spectral range.

Method	*x*	$d_{\mathrm{film}}$ (nm)	σ (S/cm)	*T* (%)	*R* (%)	T+R (%)
Sputter (Sn, ZnO)	0.4	90	180	77	23	100
Sputter (Sn, ZnO)	0.3	89	160	77	22	99
Spray pyrolysis	0.43	62	110	77	22	99

## References

[1] Granqvist C.G. (2009). Transparent conductors for solar energy and energy efficiency: a broad-brush picture. Int. J. Nanotechnol..

[2] Beyer W., Hupkes J., Stiebig H. (2007). Transparent conducting oxide films for thin film silicon photovoltaics. Thin Solid Films.

[3] Hosono H. (2007). Recent progress in transparent oxide semiconductors: Materials and device application. Thin Solid Films.

[4] Granqvist C.G., Azens A., Hjelm A., Kullman L., Niklasson G.A., Ronnow D., Stromme Mattsson M., Veszelei M., Vaivars G. (1998). Recent advances in electrochromics for smart windows applications. Sol. Energy.

[5] Fortunato E., Barquinha P., Martins R. (2012). Oxide Semiconductor Thin-Film Transistors: A Review of Recent Advances. Adv. Mater..

[6] Minami T. (2005). Transparent conducting oxide semiconductors for transparent electrodes. Semicond. Sci. Technol..

[7] Yabuta H., Sano M., Abe K., Aiba T., Den T., Kumomi H., Nomura K., Kamiya T., Hosono H. (2006). High-mobility thin-film transistor with amorphous InGaZnO_4_ channel fabricated by room temperature rf-magnetron sputtering. Appl. Phys. Lett..

[8] Jeong J.H.K.J.H., Jeong J.H.K.J.H., Yang H.W., Park J.S.J.S., Mo Y.G.Y.G., Kim H.D. (2007). High performance thin film transistors with cosputtered amorphous indium gallium zinc oxide channel. Appl. Phys. Lett..

[9] Hoel C.A., Mason T.O., Gaillard J.F., Poeppelmeier K.R. (2010). Transparent conducting oxides in the ZnO-In_2_O_3_-SnO_2_ system. Chem. Mater..

[10] Morales-Masis M., Dauzou F., Jeangros Q., Dabirian A., Lifka H., Gierth R., Ruske M., Moet D., Hessler-Wyser A., Ballif C. (2016). An Indium-Free Anode for Large-Area Flexible OLEDs: Defect-Free Transparent Conductive Zinc Tin Oxide. Adv. Funct. Mater..

[11] Kamiya T., Nomura K., Hosono H. (2010). Present status of amorphous In-Ga-Zn-O thin-film transistors. Sci. Technol. Adv. Mater..

[12] Nomura K., Ohta H., Takagi A., Kamiya T., Hirano M., Hosono H. (2004). Room-temperature fabrication of transparent flexible thin-film transistors using amorphous oxide semiconductors. Nature.

[13] Jackson W.B., Hoffman R.L., Herman G.S. (2005). High-performance flexible zinc tin oxide field-effect transistors. Appl. Phys. Lett..

[14] Rucavado E., Jeangros Q., Urban D.F., Holovský J., Remes Z., Duchamp M., Landucci F., Dunin-Borkowski R.E., Körner W., Elsässer C. (2017). Enhancing the optoelectronic properties of amorphous zinc tin oxide by subgap defect passivation: A theoretical and experimental demonstration. Phys. Rev. B.

[15] Chen Y., Lan W., Wang J., Zhu R., Yang Z., Ding D., Tang G., Wang K., Su Q., Xie E. (2016). Highly flexible, transparent, conductive and antibacterial films made of spin-coated silver nanowires and a protective ZnO layer. Phys. E Low-Dimens. Syst. Nanostruct..

[16] Göbelt M., Keding R., Schmitt S.W., Hoffmann B., Jäckle S., Latzel M., Radmilović V.V., Radmilović V.R., Spiecker E., Christiansen S. (2015). Encapsulation of silver nanowire networks by atomic layer deposition for indium-free transparent electrodes. Nano Energy.

[17] Eranna G., Joshi B.C., Runthala D.P., Gupta R.P. (2004). Oxide materials for development of integrated gas sensors - A comprehensive review. Crit. Rev. Solid State Mater. Sci..

[18] Tynell T., Karppinen M. (2014). Atomic layer deposition of ZnO: A review. Semicond. Sci. Technol..

[19] Johnson R.W., Hultqvist A., Bent S.F. (2014). A brief review of atomic layer deposition: From fundamentals to applications. Mater. Today.

[20] Kim H., Lee H.B.R., Maeng W.J. (2009). Applications of atomic layer deposition to nanofabrication and emerging nanodevices. Thin Solid Films.

[21] Kamiya T., Narushima S., Mizoguchi H., Shimizu K., Ueda K., Ohta H., Hirano M., Hosono H. (2005). Electrical properties and structure of p-type amorphous oxide semiconductor ZnO-Rh_2_O_3_. Adv. Funct. Mater..

[22] Qin P.L., Fang G.J., He Q., Sun N.H., Fan X., Zheng Q., Chen F., Wan J.W., Zhao X.Z. (2011). Nitrogen doped amorphous chromium oxide: Stability improvement and application for the hole-transporting layer of organic solar cells. Sol. Energ. Mat. Sol. Cells.

[23] Fleischer K., Caffrey D., Farrell L., Norton E., Mullarkey D., Arca E., Shvets I.V. (2015). Raman spectra of p-type transparent semiconducting Cr_2_O_3_:Mg. Thin Solid Films.

[24] Fleischer K., Norton E., Mullarkey D., Caffrey D., Shvets I.V. (2017). Quantifying the performance of P-type transparent conducting oxides by experimental methods. Materials.

[25] Norton E., Farrell L., Zhussupbekova A., Mullarkey D., Caffrey D., Papanastasiou D.T., Oser D., Bellet D., Shvets I.V., Fleischer K. (2018). Bending stability of Cu_0.4_CrO_2_ - A transparent p-type conducting oxide for large area flexible electronics. AIP Adv..

[26] Lunca Popa P., Crêpellière J., Nukala P., Leturcq R., Lenoble D. (2017). Invisible electronics: Metastable Cu-vacancies chain defects for highly conductive p-type transparent oxide. Appl. Mater. Today.

[27] Lunca-Popa P., Afonso J., Grysan P., Crêpellière J., Leturcq R., Lenoble D. (2018). Tuning the electrical properties of the p-type transparent conducting oxide Cu_1-x_Cr_1+x_O_2_ by controlled annealing. Sci. Rep..

[28] Arora A.K., Rajalakshmi M., Ravindran T.R., Sivasubramanian V. (2007). Raman spectroscopy of optical phonon confinement in nanostructured materials. J. Raman Spectrosc..

[29] Roodenko K., Goldthorpe I.A., McIntyre P.C., Chabal Y.J. (2010). Modified phonon confinement model for Raman spectroscopy of nanostructured materials. Phys. Rev. B.

[30] John N., George S. (2017). Raman Spectroscopy. Spectroscopic Methods for Nanomaterials Characterization.

[31] Beeman D., Tsu R., Thorpe M.F. (1985). Structural information from the Raman spectrum of amorphous silicon. Phys. Rev. B.

[32] Vink R.L., Barkema G.T., van Der Weg W.F. (2001). Raman spectra and structure of amorphous Si. Phys. Rev. B.

[33] Ferrari A., Robertson J. (2000). Interpretation of Raman spectra of disordered and amorphous carbon. Phys. Rev. B.

[34] Esser N. (1999). Analysis of semiconductor surface phonons by Raman spectroscopy. Appl. Phys. A Mater. Sci. Process..

[35] Fleischer K., Chandola S., Esser N., Richter W., McGilp J.F. (2003). Phonon and polarized reflectance spectra from Si(111) - (4 × 1)In: Evidence for a charge-density-wave driven phase transition. Phys. Rev. B.

[36] Liebhaber M., Bass U., Bayersdorfer P., Geurts J., Speiser E., Räthel J., Baumann A., Chandola S., Esser N. (2014). Surface phonons of the Si(111)-(7 × 7) reconstruction observed by Raman spectroscopy. Phys. Rev. B.

[37] Lieber C.A., Mahadevan-Jansen A. (2003). Automated Method for Subtraction of Fluorescence from Biological Raman Spectra. Appl. Spectrosc..

[38] Schulze H.G., Foist R.B., Okuda K., Ivanov A., Turnera R.F.B. (2012). A small-window moving average-based fully automated baseline estimation method for raman spectra. Appl. Spectrosc..

[39] Schulze H.G., Foist R.B., Okuda K., Ivanov A., Turner R.F. (2011). A model-free, fully automated baseline-removal method for raman spectra. Appl. Spectrosc..

[40] Tomba J.P., Arzondo L.M., Pastor J.M. (2007). Depth profiling by confocal Raman microspectroscopy: Semi-empirical modeling of the Raman response. Appl. Spectrosc..

[41] Zhang W.R., Lowe C., Smith R. (2009). Depth profiling of clear coil coating by confocal Raman microscopy. Prog. Org. Coat..

[42] Everall N.J. (2000). Modeling and measuring the effect of refraction on the depth resolution of confocal Raman microscopy. Appl. Spectrosc..

[43] Verre R., Svedendahl M., Shvets I.V., Odebo Länk N., Maccaferri N., Käll M., Dmitriev A., Vavassori P., Fleischer K. (2016). Polarization conversion-based molecular sensing using anisotropic plasmonic metasurfaces. Nanoscale.

[44] Zhussupbekova A., Kaisha A., Vijayaraghavan R.K., Fleischer K., Shvets I.V., Caffrey D. (2019). Importance of Local Bond Order to Conduction in Amorphous, Transparent, Conducting Oxides: The Case of Amorphous ZnSnO_y_. ACS Appl. Mater. Interfaces.

[45] Damen T.C., Porto S.P.S., Tell B. (1966). Raman effect in zinc oxide. Phys. Rev..

[46] Scott J.F. (1970). Raman Spectrum of SnO_2_. J. Chem. Phys..

[47] Eifert B., Becker M., Reindl C.T., Giar M., Zheng L., Polity A., He Y., Heiliger C., Klar P.J. (2017). Raman studies of the intermediate tin-oxide phase. Phys. Rev. Mater..

[48] Gouadec G., Colomban P. (2007). Raman Spectroscopy of nanomaterials: How spectra relate to disorder, particle size and mechanical properties. Prog. Cryst. Growth Charact. Mater..

[49] Rajalakshmi M., Arora A.K., Bendre B.S., Mahamuni S. (2000). Optical phonon confinement in zinc oxide nanoparticles. J. Appl. Phys..

[50] Wróbel J., Kurzydłowski K.J., Hummer K., Kresse G., Piechota J. (2009). Calculations of ZnO properties using the Heyd-Scuseria-Ernzerhof screened hybrid density functional. Phys. Rev. B.

[51] Lan T., Li C.W., Fultz B. (2012). Phonon anharmonicity of rutile SnO_2_ studied by Raman spectrometry and first principles calculations of the kinematics of phonon-phonon interactions. Phys. Rev. B.

[52] Bora T., Al-Hinai M.H., Al-Hinai A.T., Dutta J. (2015). Phase Transformation of Metastable ZnSnO_3_ Upon Thermal Decomposition by In-Situ Temperature-Dependent Raman Spectroscopy. J. Am. Ceram. Soc..

[53] Mauit O., Caffrey D., Ainabayev A., Kaisha A., Toktarbaiuly O., Sugurbekov Y., Sugurbekova G., Shvets I.V., Fleischer K. (2019). Growth of ZnO:Al by atomic layer deposition: Deconvoluting the contribution of hydrogen interstitials and crystallographic texture on the conductivity. Thin Solid Films.

[54] Mora-Fonz D., Shluger A.L. (2019). Making amorphous ZnO: Theoretical predictions of its structure and stability. Phys. Rev. B.

[55] Moriga T., Hayashi Y., Kondo K., Nishimura Y., Murai K.i., Nakabayashi I., Fukumoto H., Tominaga K. (2004). Transparent conducting amorphous Zn–Sn–O films deposited by simultaneous dc sputtering. J. Vac. Sci. Technol. A Vac. Surfaces Film..

[56] Yue S., Lu J., Lu R., Li S., Li X., Zhang J., Chen L., Ye Z. (2018). Ultrathin amorphous ZnGexSnO films for high performance ultra-thin-film transistors. Appl. Phys. Lett..

[57] Korepanov V.I., Chan S.Y., Hsu H.C., Hamaguchi H.o. (2019). Phonon confinement and size effect in Raman spectra of ZnO nanoparticles. Heliyon.

[58] Montenegro D.N., Hortelano V., Martínez O., Martínez-Tomas M.C., Sallet V., Muñoz-Sanjosé V., Jiménez J. (2013). Non-radiative recombination centres in catalyst-free ZnO nanorods grown by atmospheric-metal organic chemical vapour deposition. J. Phys. D. Appl. Phys..

